# Refining Adjuvant Therapy for Endometrial Cancer: New Standards and Perspectives

**DOI:** 10.3390/biology10090845

**Published:** 2021-08-30

**Authors:** Alessandra Giustozzi, Vanda Salutari, Elena Giudice, Lucia Musacchio, Caterina Ricci, Chiara Landolfo, Maria Teresa Perri, Giovanni Scambia, Domenica Lorusso

**Affiliations:** 1 Institute of Obstetrics and Gynecology, Università Cattolica del Sacro Cuore, Largo Agostino Gemelli 8, 00168 Rome, Italy; alessandra.giustozzi01@icatt.it (A.G.); elenagiudice6@gmail.com (E.G.); mariateresa.perri91@gmail.com (M.T.P.); giovanni.scambia@policlinicogemelli.it (G.S.); 2 Department of Woman, Child and Public Health, Fondazione Policlinico Universitario A. Gemelli IRCCS, Largo Agostino Gemelli 8, 00168 Rome, Italy; vanda.salutari@policlinicogemelli.it (V.S.); luciamusacchio89@gmail.com (L.M.); caterina.ricci@policlinicogemelli.it (C.R.); chiara.landolfo@policlinicogemelli.it (C.L.)

**Keywords:** endometrial carcinoma, molecular markers, guidelines, treatment

## Abstract

**Simple Summary:**

In recent years, the adjuvant treatment of endometrial carcinoma has changed due to the integration of the molecular features in the clinical–pathological classification. The new ESGO/ESTRO/ESP guidelines (2021) proposed the evaluation of the adjuvant treatment of endometrial carcinoma using a prognostic-risk group stratification based on pathogenetic, clinical, and molecular features. Moreover, the adjuvant therapy of endometrial carcinoma is currently being re-defined in ongoing studies. This review provides a comprehensive overview of endometrial carcinoma adjuvant therapy, analyzing the “new standards” and “new perspectives”.

**Abstract:**

Endometrial carcinoma is the most frequent cancer of the reproductive female organs. Most endometrial cancers are diagnosed at early stage (75%). Treatment options depend on pathogenetic, histopathologic and clinical characteristic at the diagnosis. To improve patient management in the near future, recent research has focused on new molecular features; evidence has shown that these give a better definition of patient prognosis and can help in tailoring adjuvant treatments by identifying specific subgroups of patients whose tumors may benefit from specific therapeutic approaches. In this review, we will focus on current knowledge of adjuvant treatment of endometrial carcinoma, using a prognostic-risk group stratification based on pathogenetic, clinical and molecular features, and will take a look at the ongoing trials that will further change the therapeutic approach in coming years.

## 1. Introduction

Endometrial carcinoma (EC) is the fourth most frequently diagnosed cancer in women with 130,051 estimated new cases and 29,963 deaths in Europe in 2020 [1]. It is the most common gynecologic malignancy in the developed countries. Most endometrial cancers are diagnosed at early stage (75%), and the reported survival rate is 75%. The incidence of this neoplasm tends to increase with population aging: in 15–44 years-old women EC is extremely rare, with 4729 new cases in 2020, whereas in women over 45, the incidence increases up to 125,321 new cases [1]. In the last 20 years an increase in endometrial cancer incidence over time has been reported, possibly related to the decreased use of approved estrogen–progestogen therapy associated with the increase in compounded bioidentical hormone therapy (CBHT) use, and the prevalence of obesity and diabetes [2]. The five-year overall survival (OS) of women diagnosed with uterine cancer was estimated at 76% in the Eurocare 5-study [3].

Over the years, EC has been classified using three different approaches: pathogenetic, histopathologic and molecular. The first was proposed by Bokhman in 1983. He classified two clinic-pathological types [4]; type I endometrial carcinoma is more frequent in perimenopausal age, is associated with exogenous or endogenous estrogen stimulation and is mainly represented by low-grade endometrioid carcinomas. Type II is more frequent in menopausal age; it is not related to estrogen stimulation and is enriched by serous and clear cell carcinomas [5]. The fifth edition of the World Health Organization (WHO)’s histopathological classification is currently used for the diagnosis of endometrial carcinoma and is based on microscopic features: endometroid carcinoma and its variants, mucinous carcinoma, serous carcinoma, neuroendocrine tumors, mixed-cell adenocarcinoma, and undifferentiated and dedifferentiated carcinoma; this classification includes both histopathological features and molecular class [6].

The Cancer Genome Atlas (TCGA) performed an integrated genomic and proteomic analysis of 373 endometrial cancers that provided insights into disease biology and diagnostic classification with immediate therapeutic application [7]. It identified four molecular subgroups: DNA polymerase epsilon (POLE)-mutated (ultra-mutated, (POLEmut)), microsatellite unstable (hypermutated), copy number low, and copy number high. This new classification requires the performance of copy-number analysis, which is not feasible in routine clinical practice; however, surrogate markers have been developed and validated to arrive at an assessable classification. [8,9,10] As performing an isolated surrogate marker test is insufficient, a diagnostic algorithm to classify ‘multiple classifier’ tumors has been developed [11,12]. In addition, endometrial carcinoma could be classified as POLEmut if any pathogenic variants of POLE were identified in the gene’s exonuclease domain [13]. In this context, ECs analogous to the somatic copy number alteration (SCNA)-high subclass can be identified by p53 immuno-histochemistry (p53-mutated (p53abn) EC) [14], the microsatellite instability (MSI) subclass by immunohistochemistry for mismatch repair (MMR) proteins (mismatch repair deficient (MMRd) EC), and the POLE subclass by targeted sequencing of the POLE exonuclease domain (POLEmut EC). Tumors lacking these three prior features are classified as p53 wild-type (p53wt EC) [10] or no specific molecular profile subtype (NSMP EC) [11], analogous to the SCNA-low subclass. This surrogate marker approach allows professionals to classify most ECs into one molecular class. There are also ‘multiple-classifiers’, i.e., tumors that have more than one molecular classifying feature, that represent 3–6% of ECs. These include tumors with combined POLE exonuclease domain mutation (EDM) and their correlated p53abn, tumors with combined MMRd and their correlated p53abn, tumors with combined MMRd and EDM (MMRd-POLEmut) and, finally, all three: MMRd–POLEmut–p53abn. When p53 mutations occur in POLEmut or MMRd ECs, they are not associated with a worst prognosis so that they are considered passenger mutations, i.e., a later event during tumor progression in multiple-classifier EC, and the molecular behaviour or the phenotype of tumors with both POLEmut and p53abn, or MMRd and p53abn, is that of ECs with POLEmut or MMRd, respectively. For clinical practice, this means that the presence of p53 mutations in the context of MMRd EC or POLEmut EC does not determine the need for more intensive treatment [8,9,10,11,13]. A recent study suggests that tumors with any of the 11 POLE EDMs identified in the TCGA should be classified as POLE-mut EC, independently of MMRd/MSI and p53 status [15].

## 2. Adjuvant Treatment for Endometrial Carcinoma according to the Prognosis Risk Groups

The new European Society of Gynaecological Oncology/European Society for Radiotherapy & Oncology/European Society of Pathology (ESGO/ESTRO/ESP) guidelines for the management of patients with endometrial carcinoma, defined in 2021, give new recommendations for adjuvant treatments which are strongly dependent on the prognostic risk group, defined according to several elements: 1. histopathologic type, according to WHO Classification of tumors (5th edition) [6]; 2. grade, using a binary International Federation of Gynecology and Obstetrics (FIGO) grading, which considers grade 1 and grade 2 carcinomas as low-grade and grade 3 carcinomas as high-grade; 3. myometrial invasion and 4. lympho-vascular space involvement (LVSI) [16]. A broader and comprehensive vision of tumor features is, at present, possible thanks to the molecular classification that adds new information to the previous knowledge [17] and that, for the first time, has been integrated into the new guidelines.

In this review we will focus on the adjuvant treatment proposed for each prognostic risk group of EC in the new ESGO/ESTRO/ESP Guidelines and review the literature supporting the final statements. Risk groups with and without molecular classification are described in Table 1.

### 2.1. Low Risk (LR)

The Low-risk group includes stage IA endometrioid carcinoma, low-grade and LVSI negative or focal (defined by the presence of a single focus around the tumor). A consensus has been found for managing these tumors: no adjuvant treatment is recommended. The data backing this conclusion are based mainly on multiple randomized trials reporting no improvement in prognosis when adjuvant RT is given, at the cost of an increased toxicity [18,19,20,21].

The sub-group analysis of the PORTEC-3 trial showed that patients with POLEmut EC have an excellent prognosis independently of adjuvant treatment choice [8,11,22]. Based on this, the new ESGO/ESTRO/ESP guidelines do not recommend adjuvant treatment for patients with stage I-II EC with POLE mutation. However, scientific evidence is not currently able to support the effects of the omission of adjuvant treatment for the small number of patients with stage III-IVA, POLEmut EC. Therefore, prospective registration in registries is recommended [17].

### 2.2. Intermediate Risk (IR)

The vagina is the most frequent site of recurrence after surgery in intermediate risk EC patients. Many randomized trials have shown that vaginal brachytherapy (VBT) is effective in preventing vaginal relapse [23,24,25,26,27,28,29,30]. A Dutch open-label, non-inferiority, randomized trial compared the outcomes and adverse effects of VBT or External Beam Radiotherapy (EBRT) as adjuvant treatment of high-intermediate risk EC. The study showed that not only does VBT successfully avoid vaginal relapse, but it also tends to have fewer gastrointestinal toxic effects than EBRT, with better quality of life [31]. In view of this, the new guidelines recommend VBT as adjuvant treatment for patients with intermediate risk EC [17] to decrease vaginal recurrence without any impact on overall survival [20].

Moreover, new standards suggest that in younger than 60-year-old IR patients the omission of adjuvant brachytherapy can be considered [17]. This is supported by the long term follow up of Alders’ trial evaluating the consequence of postoperative radiotherapy in low and intermediate-risk patients and reporting an increased mortality due to secondary, radiotherapy-induced tumors in younger patients [20].

When the molecular classification is known, into this category are placed stage IA p53abn and/or non-endometrioid (serous, clear cell, undifferentiated carcinoma, carcinosarcoma, mixed) without myometrial invasion, and MMRd and NMSP EC with IR clinical and pathologic characteristics.

In this setting, published data are very scarce; however, according to some case series, vaginal brachytherapy might be effective in preventing vaginal relapse in stage I type 2 endometrial cancer, while others have suggested that adjuvant chemotherapy could improve survival [23]. Based on this apparently contradictory result, the expert panel concluded that the management of these patients should be discussed on a case-by-case basis, as surrounding evidence is not sufficient [17].

Lastly, in the case of P53abn carcinoma restricted to a polyp or without myometrial invasion, there are no available randomized trials and new standards do not recommend adjuvant therapy due to the uncertainty of its effect [17].

### 2.3. High-Intermediate Risk (HIR)

The long-term, 10-year follow up analysis of the PORTEC-2 trial reported that substantial LVSI and L1 Cell Adhesion Molecule (L1CAM) expressions are strong predictive risk factors for recurrence in intermediate risk EC patients. In the PORTEC-2 trial, women with these unfavorable risk factors showed a greater risk of locoregional recurrence: based on this new evidence another risk group has been defined (high-intermediate risk) for which EBRT is recommended [32].

As for the management of this risk group we need to differentiate two different clinical situations: 1. when lympho-nodal status is known and negative (patients received lymphadenectomy or sentinel lymph node procedure): new guidelines recommend adjuvant brachytherapy to reduce vaginal recurrence; 2. when the lympho-nodal status is unknown because staging was not performed: EBRT is the treatment of choice. Chemotherapy can be considered especially for high grade and/or substantial LSVI [17].

The first evidence of a possible role for adjuvant chemotherapy in HIR EC was reported by Maggi et al. [33] and Susumu et al. [34]. Maggi’s trial was a randomized phase 3 trial comparing adjuvant EBRT with 5 cycles of cyclophosphamide–doxorubicin–cisplatin (CAP) chemotherapy (CT) in women with endometrioid FIGO stage IC G3, IIa-b G3 with deep myometrial invasion or stage III carcinoma. Despite the fact that the study did not show any differences in progression-free survival (PFS) and OS between CT and radiotherapy (RT), multivariate analysis showed that age, grading, depth of myometrial invasion, and FIGO stage were all significantly associated with PFS and OS [33].

Susumu’s study is a multi-center randomized phase III trial comparing EBRT with 3 cycles of CAP in women with endometrioid adenocarcinoma with deeper than 50% myometrial invasion. The observed PFS and OS were not statistically different between the two regimens, but a subgroup analysis showed that CT significantly improved PFS and OS in HIR patients with respect to EBRT [34]. Both the trials reported that radiation treatment reduces loco-regional recurrences, while CT was associated with a better systemic control of disease. The logical consequence of this observation was to combine the two strategies.

The combined analysis of NSGO/EORTC-trials showed that the sequential combination of CT and EBRT was associated with a 36% reduction in the risk of relapse or death and a 49% reduction in the risk of cancer-related death [35]. The NSGO-9501/EORTC-55991 trial included patients with FIGO stage I-II, IIIA (only positive peritoneal fluid cytology) and stage IIIC (only positive pelvic lymph nodes without postoperative macroscopic residual tumor) EC. The MaNGO ILIADE- III study included patients with endometrioid carcinoma FIGO stage IIB, IIIA–C (stage IIIA with positive cytology alone, without other risk factors, was not included). It has to be noted that these trials had some limitations, including the eligibility of a heterogeneous population with respect to risk factors, the majority of which were at low risk, which may have impacted the statistical power of the trial [35]. However, the five-year survival rates in the control arm were consistent with historical data [33,34].

More recently the results of the GOG 249 trial [36] were reported. The study is a randomized phase III trial evaluating EBRT vs. 3 cycles of platinum–paclitaxel chemotherapy plus or minus VBT in HIR and high-risk (HR) early-stage EC. The study showed that the postoperative adjuvant strategy of VBT followed by CT was not a better course than EBRT in terms of recurrence free survival and was also associated with more frequent and severe acute toxicity [36]. The published randomized trial’s demonstrated high-intermediate risks are listed in Table 2. Concerning the HIR category, currently we are waiting for the PORTEC-4a trial results, which represents the first ongoing trial comparing the molecular classifier against conventional histopathological risk groups [37].

With a lower level of evidence, suggesting a general uncertainty about the best treatment for these patients, other options were reported by the panel, which stated that a “tailored” treatment may be the better choice for HIR EC patients: omission of any adjuvant treatment and adjuvant brachytherapy alone, particularly for high-grade LVSI negative and for stage II grade 1 ECs, are also reported options [17]. When molecular classification is known, placed in this category are stage I MMRd/NSMP endometrioid carcinoma with substantial LVSI, regardless of grade and depth of invasion, stage IB MMRd/NSMP endometrioid carcinoma high-grade, regardless of LVSI status and stage II MMRd/NSMP endometrioid carcinoma.

The molecular sub-group analysis of PORTEC-3 trial suggests that there is no benefit from CT in MMRd carcinomas [22,38].

### 2.4. High Risk (HR)

For these patients the recommendations are represented by EBRT with concurrent and adjuvant CT, or in alternative sequential CT and radiotherapy. Chemotherapy alone is also an option [17].

The role of combined adjuvant chemotherapy and concurrent chemo-radiotherapy is reported in the PORTEC 3 trial, which compared this strategy with EBRT alone in high-risk EC. Updated results of this trial, published in 2019 when the majority of the patients had reached 5 years of follow-up, showed improved 5-year OS (81.4% versus 76.1% with chemoradiotherapy and radiotherapy alone, respectively) and failure-free survival (76.5% versus 69.1% with CT and RT, respectively). In subgroup analysis, women with stage III endometrial carcinomas and serous carcinomas reported the greatest advantage while, for women with stage I–II endometrial cancer, combined adjuvant treatment translated only to a small absolute improvement of 2% in 5-year OS and 4% in failure-free survival [39], thus suggesting that for stage I-II patients the option of adding CT to radiotherapy should be discussed in the light of the risk/benefit assessment.

The GOG-258 study was a randomized phase III trial evaluating six cycles of platinum-paclitaxel CT vs. tumor-directed RT followed by CT in patients with stage III-IVA endometrial cancer. This trial showed that the combined regimen was not superior to chemotherapy alone in prolonging relapse-free survival, although locoregional relapses were less frequent than with chemotherapy alone. [40].

When the molecular classification is known, the subgroup analysis of the PORTEC-3 trial reported an improved OS from combined therapy for stage I-III p53abn carcinomas with myometrial infiltration; benefits of CT were, however, not clear for MMRd carcinomas, while stage III NSMP carcinomas seemed to see some benefit from the addition of chemotherapy.

## 3. Guidelines Gap: What Needs to Be Investigated

Despite the recent implementation of molecular features into clinical classification in order to better individualize the adjuvant treatment of endometrial cancer, there are still some limitations to be addressed and some settings for which the 2021 guideline does not provide strong recommendations, due to a lack of a high level of evidence [17]. Table 3 summarizes this “guidelines gap” in order to underline the clinical needs of randomized trials to define the most appropriate treatment for these subgroups. Moreover, it needs to be underlined that the present recommendation refers to retrospective subgroup analysis of the PORTEC studies, prospective confirmation of this data being still lacking.

## 4. Discussion

Next perspectives for the adjuvant therapy of endometrial carcinoma are currently being defined in ongoing studies.

The RAINBO trial is an international program of personalized, molecular based, adjuvant treatment for high-risk EC patients. The objective of the program is to improve the management of endometrial carcinoma by approaching the cure with a molecular-driven strategy and to compare standard treatment to personalized treatment based on molecular features. After surgery, EC patients will be recruited in four independent and parallel trials based on their molecular profiling: P53abn, MMRd, NSMP and POLEmut. The p53abn-RED trial will investigate the role of 2-year maintenance therapy with Niraparib after chemo-radiotherapy in stage I-III p53abn ECs. The MMRd-GREEN trial will evaluate the role of adjuvant anti PD-L1 inhibitors in stage II-III MMRd EC with substantial LVSI. The NSMP-ORANGE trial will compare the addition of hormone adjuvant therapy to EBRT vs. the addition of CT to EBRT in stage II-III NSMP EC patients. The POLEmut-BLUE trial is a single arm prospective phase II trial of observation for all stages POLEmut EC; adjuvant treatment is not planned, with the exception of EBRT in advanced stage.

The role of immunotherapy in combination with CT in the adjuvant setting of high-risk EC is being addressed in the ENGOT-EN11 ongoing trials. This is a randomized phase 3 trial evaluating the role of anti PD-1 Pembrolizumab in combination with platinum-paclitaxel chemotherapy vs. CT/placebo in newly diagnosed endometrial FIGO stage I/II non-endometrioid histology, FIGO Stage I/II any histology with known p53abn expression and FIGO Stage III/IVA any histology carcinoma or carcinosarcoma.

Patient should have received curative intent surgery with no residual disease and no prior radiation or systemic therapy for endometrial cancer including neoadjuvant therapy. RT is optional in both arms. The primary end points of the trial are both disease-free-survival (DFS) and OS.

It is expected that these trials, if positive, will change the adjuvant treatment strategy of EC and the molecular features will guide in the next future the choice of treatment, with a more targeted treatment approach.

It has also to be noticed that, in the individualized therapies era, other targeted agents are under investigation. In the adjuvant setting, antibodies targeting HER2 over-expression within the p53abn group have shown promising results [41].

## 5. Conclusions

The integration of molecular features in the clinical–pathological classification of EC represents an urgent step forward and definitive turning point in the general management of the disease. The possibility of identifying the molecular characteristics of patients will offer the opportunity to better define the prognosis of the disease and to individualize treatments according to well defined molecular profiling.

## Figures and Tables

**Table 1:** Endometrial cancer classification.

	Low Risk	Intermediate Risk	High-Intermediate Risk	High Risk
**Histopathological** **and Clinical** **classification**	Stage IA endometrioid + low-grade + LVSI negative or focal	Stage IB endometrioid + low-grade+LVSI negative or focal	Stage I endometrioid+ substantial-LVSI, regardless of grade and depth of invasion	Stage III-IVA with no residual disease
		Stage IA endometrioid + high-grade+LVSI negative or focal	Stage IB endometrioid high-grade, regardless of LVSI status	Stage I-IVA non-endometrioid (serous, clear cell, undifferentiated carcinoma, carcinosarcoma, mixed) with myometrial invasion, and with no residual disease
		Stage IA non-endometrioid (serous, clear cell, undifferentiated carcinoma, carcinosarcoma, mixed) without myometrial invasion	Stage II	
**Molecular Classification** **Known**	Stage I-II POLEmut endometrial carcinoma, no residual disease	Stage IB MMRd/NSMP endometrioid carcinoma + low-grade + LVSI negative or focal	Stage I MMRd/NSMP endometrioid carcinoma + substantial LVSI, regardless of grade and depth of invasion	Stage III-IVA MMRd/NSMP endometrioid carcinoma with no residual disease
	Stage IA MMRd/NSMP endometrioid carcinoma + low-grade + LVSI negative or focal	Stage IA MMRd/NSMP endometrioid carcinoma + high-grade + LVSI negative or focal	Stage IB MMRd/NSMP endometrioid carcinoma high-grade, regardless of LVSI status	Stage I-IVA p53abn endometrial carcinoma with myometrial invasion, with no residual disease
		Stage IA p53abn and/or non-endometrioid (serous, clear cell, undifferentiated carcinoma, carcinosarcoma, mixed) without myometrial invasion	Stage II MMRd/NSMP endometrioid carcinoma	Stage I-IVA NSMP/MMRd serous, undifferentiated carcinoma, carcinosarcoma with myometrial invasion, with no residual disease

**Table 2:** Randomized trial concerning high-intermediate risk.

**R Maggi** **et al.** [33]	A randomized controlled trial	A total of 345 patients were randomly assigned; 168 to external RT and 177 to adjuvant CT	To evaluate whether adjuvant CT confers an advantage for overall and progression-free survival and on the incidence of local and distant relapses over standard pelvic RT, in high-risk patients without residual tumor.	First evidence of the possibility to combine RT and CT.	No improvement in PFS and OS in patients treated with one or the other treatment protocol. Both therapeutic approaches were associated with acceptable toxicities.
**Nobuyuki Susumu** **et al.** [34]	A randomized phase III trial	A total of 385 patients were randomly assigned; 193 to pelvic radiation therapy (PRT) and 192 to cyclophosphamide–doxorubicin–cisplatin (CAP) chemotherapy.	To establish an optimal adjuvant therapy for intermediate- and high-risk endometrial cancer patients.	Adjuvant chemotherapy may be a useful alternative to radiotherapy for intermediate-risk endometrial cancer.	No statistically significant differences in survivals in the two regimens. Adverse effects were not significantly increased in a platinum-based combined chemo- therapy group. Chemotherapy significantly improved PFS and OS in HIR patients, versus pelvic radiation.
**Thomas Hogberg et al.** [35]	Two randomized trial	A total of 383 patients were randomly assigned; 183 to RT and 187 to RT-CT; a total of 157 patients were randomly assigned; 76 to RT and 80 to RT-CT.	To evaluate if sequential combination of chemotherapy and radiotherapy improves progression-free survival (PFS) in high-risk endometrial cancer.	The sequential addition of CT to RT was associated with a significant 36% reduction in the risk of relapse or death and a significant 49% reduction.	Addition of adjuvant chemotherapy to radiation improves progression-free survival in operated endometrial cancer patients with no residual tumor and a high-risk profile.
**Marcus E.** **Randall et al.** [36]	A randomized phase III trial	A total of 601 patients were randomly assigned; 301 to PRT and 300 to vaginal cuff brachytherapy plus three cycles of carboplatin and paclitaxel repeated every 3 weeks.	To determine if vaginal cuff brachytherapy and chemotherapy (VCB/C) increases recurrence-free survival (RFS) compared with PRT in high-intermediate and high-risk early-stage endometrial carcinoma.	Post-operative adjuvant therapy with VCB/C was not superior to EBRT and was associated with more frequent and severe acute toxicity.	Pelvic RT remains an appropriate treatment for high-risk early-stage endometrial carcinoma

**Table 3:** Lack of evidence in endometrial cancer guidelines.

Endometrial Cancer Classification	Guideline Recommendations	Levels of Evidence
LR	Stage I–II with POLE-mut: omission of adjuvant treatment	IIIA
	Stage III–IVa with POLE-mut: omission of adjuvant treatment	IVC
IR	Omission of brachytherapy considered	IIIC
	p53abn without myometrial invasion/polyp: omission of adjuvant treatment	IIIC
HIR	pN0 after lymph node staging: omission of adjuvant treatment	IVC
HR	Carcinosarcoma considered as HR carcinomas (not as sarcomas)	IVC

## Data Availability

Not applicable.
